# Lactic acid bacteria and bifidobacteria deliberately introduced into the agro-food chain do not significantly increase the antimicrobial resistance gene pool

**DOI:** 10.1080/19490976.2022.2127438

**Published:** 2022-09-28

**Authors:** Vita Rozman, Petra Mohar Lorbeg, Primož Treven, Tomaž Accetto, Majda Golob, Irena Zdovc, Bojana Bogovič Matijašić

**Affiliations:** a University of Ljubljana, Biotechnical Faculty, Department of Animal Science, Institute of Dairy Science and Probiotics, Domžale, Slovenia; b University of Ljubljana, Biotechnical Faculty, Department of Microbiology, Chair of Microbial Diversity, Microbiomics and Biotechnology, Ljubljana, Slovenia; c University of Ljubljana, Veterinary Faculty, Institute of Microbiology and Parasitology, Ljubljana, Slovenia

**Keywords:** Lactic acid bacteria, bifidobacteria, probiotic, starter culture, antimicrobial resistance, resistance gene, mobile genetic element, metagenomes, whole genome sequences

## Abstract

Lactic acid bacteria (LAB) and bifidobacteria may serve as reservoirs of antimicrobial resistance, but the risk posed by strains intentionally introduced into the agro-food chain has not yet been thoroughly investigated. The aim of our study was to evaluate whether probiotics, starter and protective cultures, and feed additives represent a risk to human health. In addition to commercial strains of LAB and bifidobacteria, isolates from human milk or colostrum, intestinal mucosa or feces, and fermented products were analyzed. Phenotypic susceptibility data of 474 strains showed that antimicrobial resistance was more common in intestinal isolates than in commercial strains. Antimicrobial resistance genes (ARGs) and mobile genetic elements (MGEs) were characterized in the whole genome sequences of 1114 strains using comparative genomics. Intrinsic ARGs were abundant in enterococci, bifidobacteria, and lactococci but were considered non-risky due to the absence of MGEs. The results revealed that 13.8% of commercial strains contained acquired ARGs, most frequently for tetracycline. We associated 75.5% of the acquired ARGs with known or novel MGEs, and their potential for transmission was assessed by examining metagenomic sequences. We confirmed that ARGs and MGEs were not as abundant or diverse in commercial strains as in human intestinal isolates or isolates from human milk, suggesting that strains intentionally introduced into the agro-food chain do not pose a significant threat. However, attention should be paid especially to individual probiotic strains containing elements that have been shown to have high potential for transferability in the gut microbiota.

**Abbreviations:** ARG, antimicrobial resistance gene; ICE, integrative and conjugative element; IME, integrative and mobilizable element; LAB, lactic acid bacteria; MDR, multidrug resistance; MIC, minimum inhibitory concentration; MGE, mobile genetic element; TRRPP, tetracycline-resistant ribosomal protection protein; WGS, whole genome sequences

## Introduction

Antimicrobials (antibiotics) have become one of the foundations of modern medicine, but the rapid spread of resistance in pathogenic bacteria threatens their effectiveness. In recent years, there is increasing evidence that lactic acid bacteria (LAB) and bifidobacteria may act as vectors for the transmission of antimicrobial resistance genes (ARGs) in the gut microbiota.^1^ The Qualified Presumption of Safety (QPS) requirement for the absence of acquired ARGs in bacteria intentionally introduced into the agro-food chain^2^ also applies to commercial strains of LAB and bifidobacteria used as starter or protective cultures, probiotics, or feed additives, which must be confirmed in their whole genome sequences since 2018.^3^ Recent studies on type strains of lactobacilli^4^ and bifidobacteria,^5^ enterococci of various origins,^6^ as well as limited number of commercial strains^7–9^ have shown the usefulness of this approach, but a comprehensive study focusing on this group of bacteria has not yet been conducted. Acquired ARGs are often carried by mobile genetic elements (MGEs), which are better characterized in enterococci^10^ than in other genera of LAB or bifidobacteria, but the potential for transmission of these elements in the gut microbiota is largely unknown. Taken together, current data are insufficient to answer with certainty the question of the safety of commercially available LAB and bifidobacteria with respect to antimicrobial resistance.

Furthermore, to strengthen the resilience of the food and agriculture sector by limiting the emergence and spread of antimicrobial resistance, it is of utmost importance to establish a solid link between the regional agri-food system and cross-sectoral interventions, as well as other stakeholders such as farmers, producers, fishermen, and physicians.^11^

To this end, present study was designed to provide a basis for assessing the risk of antimicrobial resistance of strains deliberately introduced into the agro-food chain. Four groups of LAB and bifidobacteria were analyzed at two different levels. Phenotypic susceptibility to antimicrobials was determined *in vitro* by microdilution, while whole genome sequences, mainly those from public databases, were investigated *in silico* for ARGs and MGEs. The significance and transmission potential of these elements were assessed by examining metagenomic sequences of the human gut microbiota. Our study provides new insights into the distribution of known ARGs and known and novel MGEs in commercial strains of LAB and bifidobacteria and offers new perspectives on the safety of these strains.

## Results

### Antimicrobial susceptibility profiles

**Figure 1.**
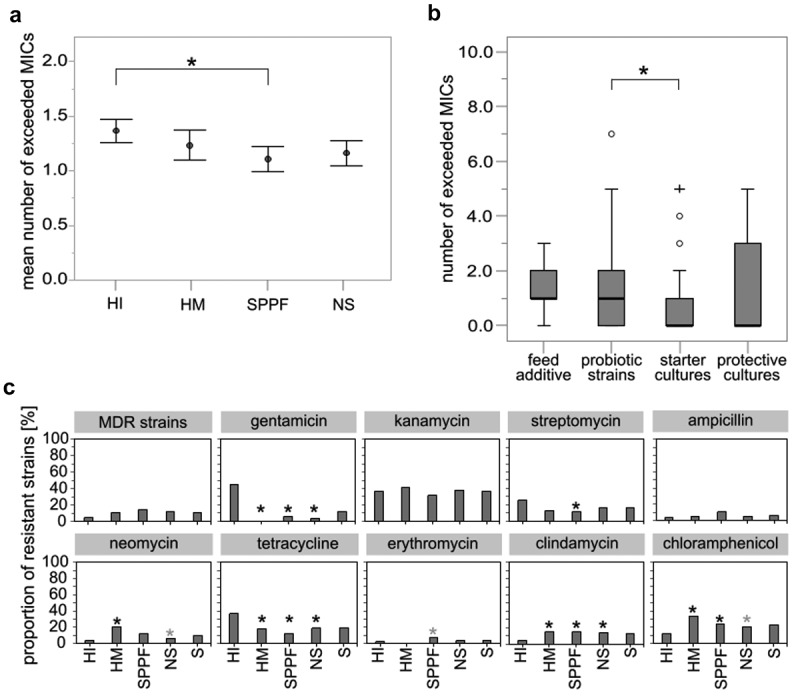
Differences in phenotypic resistance between groups of lactic acid bacteria and bifidobacteria of different origin. (a) Mean number (± standard error) of minimum inhibitory concentrations (MICs) exceeded in four groups of bacteria. (b) The number of MICs exceeded in the SPPF group. Statistics: rank analysis of variance (Kruskall-Wallis), pairwise multiple comparisons using Dunn’s test with Bonferroni adjustment; p < .05; *, statistically significant difference. (c) The proportion of phenotypically resistant strains to antimicrobials. Statistics: likelihood ratio test, Fisher’s exact probability test; p < .05; black asterisk, statistically significant difference compared to the human intestinal mucosa or feces group (HI); gray asterisk, statistically significant difference compared to the human milk or colostrum group (HM). MDR, multidrug resistance; NS, isolates of the natural microbiota from fermented products (nonstarter strains); SPPF, strains intentionally introduced into the agro-food chain via starter, protective, and probiotic cultures and feed additives; S, sum.

Phenotypic antimicrobial susceptibility of 474 isolates was determined, of which 157 belonged to the group of commercial strains (starter cultures, protective cultures, probiotics, feed supplements), 154 of which were nonstarter strains, 90 isolates from human intestinal mucosa or feces, and 73 isolates from human milk or colostrum. We obtained 4115 minimum inhibitory concentrations (MICs) of antimicrobials representing the majority of clinically important antimicrobial groups.^3^ As cutoff MICs for some antimicrobials are not available for all species,^3^ the results for 3885 MICs could only be used to classify strains as resistant or sensitive (Supplementary Table S1). The results indicate a moderate average level of resistance ˗ 14.6% of MICs were classified as resistant when all MICs were considered. Resistance was most prevalent in the human intestinal group (17.1%), whereas it was less common in the commercial strains (strains from starter and protective cultures, dietary supplements, and feed additives; 13.3%), with starter cultures exhibiting lower resistance rate (9.7%) than probiotic strains (15.7%). Statistical analysis showed a significant difference in the mean number of MICs indicating resistance between intestinal strains and commercial strains (p = .014) (Figure 1a) and between starter cultures and probiotic strains (p = .038) (Figure 1b). Conversely, no significant differences were observed in the proportion of isolates that exhibited multidrug resistance (MDR), defined as resistance to three or more classes of antimicrobials^12^ (Figure 1c). While 36.9% of isolates showed pan-susceptibility, 11.2% of isolates were MDR. Pediococci and *Levilactobacillus brevis* represented a large proportion of MDR strains. Pair by pair comparisons of the mean numbers of MICs indicating phenotypic resistances showed statistically significant differences for 24 genus pairs, mainly on the account of *Streptococcus, Levilactobacillus*, and *Pediococcus* (Supplementary Table S2). Namely, *Streptococcus* is the genus where the fewest MICs exceeded the cutoff values, while in *Levilactobacillus* and *Pediococcus* the highest number of MICs exceeding the breakpoints for resistance was observed in comparison with other genera.

As shown in Figure 1c, strains from the human intestinal group had a greater proportion of gentamicin and tetracycline resistance, whereas the opposite was observed with chloramphenicol and clindamycin. Resistance to neomycin and chloramphenicol occurred more frequently in the human milk group. Overall, microdilution tests revealed that resistance to kanamycin (36.4% of strains), chloramphenicol (22.4%), and tetracycline (19.8%) was most common, whereas the proportion of isolates resistant to erythromycin (4.0%) and ampicillin (6.5%) remained low. No vancomycin resistance was observed.

The distribution of MICs is shown in Supplementary Figure S1. In bifidobacteria, a bimodal distribution, indicative of acquired resistance,^13^ was observed for tetracycline, erythromycin, and clindamycin, whereas in lactococci it was also observed for aminoglycosides (gentamicin, kanamycin, streptomycin, neomycin). *Leuconostoc* isolates exhibited susceptibility to five groups of antimicrobials but showed presumed acquired resistance to kanamycin, neomycin, and clindamycin (Supplementary Table S1). On the other hand, a large proportion of pediococci displayed resistance to several antimicrobials with a unimodal distribution of MICs. Consistent with considerable evidence of acquired resistance in enterococci,^10^ bimodal or multimodal distributions of MICs were noted for each antimicrobial tested, with the exception of vancomycin. Surprisingly, resistance in *Streptococcus thermophilus* and *Staphylococcus* sp. was detected at a very low frequency (Supplementary Table S1).

The former genus *Lactobacillus* has recently been reclassified into 25 genera due to its extreme diversity,^14^ but still antimicrobial breakpoint values have not yet been updated to reflect the new genera. A wide range of responses to different antimicrobials was observed (see Supplementary Figure S1, genera/groups with more than 20 strains analyzed are shown). Briefly, resistance and bimodal MIC distribution were observed for clindamycin and chloramphenicol in the *Lactobacillus acidophilus* group, for streptomycin, tetracycline, erythromycin, and clindamycin in *Lacticaseibacillus rhamnosus*, for tetracycline and ampicillin in *Limosilactobacillus reuteri*, and for clindamycin in *Lactiplantibacillus plantarum*. In contrast, concomitant unimodal distribution of MICs of aminoglycosides, chloramphenicol, and/or ampicillin was observed for some species (Supplementary Figure S1).

### Antimicrobial resistance genes

**Figure 2.**
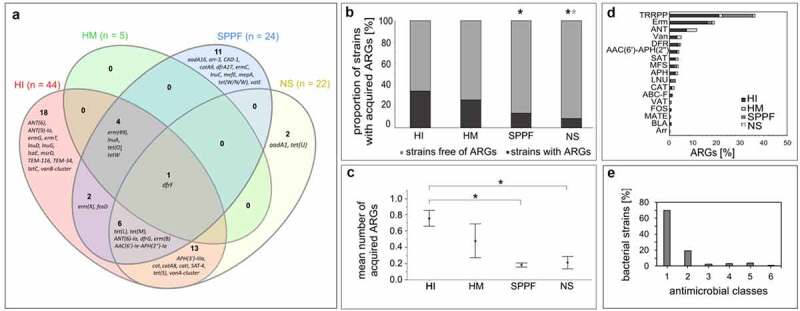
Acquired antimicrobial resistance genes (ARGs) in lactic acid bacteria and bifidobacteria of different origin. (a) Venn diagram showing acquired ARGs in four groups of bacteria. (b) The proportion of strains with acquired ARGs. Statistics: Likelihood ratio test, Fisher’s exact probability test; p < .05; black asterisk, statistically significant difference compared to the human intestinal mucosa or feces group (HI); gray asterisk, statistically significant difference compared to the human milk or colostrum group (HM). (c) The mean number (± standard error) of acquired ARGs. Statistics: rank analysis of variance (Kruskall-Wallis), pairwise multiple comparisons using Dunn’s test with Bonferroni adjustment; p < .05; *, statistically significant difference. (d) The prevalence of the protein families of acquired ARGs. (e) The prevalence of the strains according to the number of antimicrobial classes toward which the acquired ARGs confer resistance. NS, isolates of the natural microbiota from fermented products (nonstarter strains); SPPF, strains intentionally introduced into the agro-food chain via starter, protective, and probiotic cultures and feed additives; AAC(6’)-APH(2”), aminoglycoside acetyltransferase/phosphotransferase; ABC-F, ABC-F subfamily of ATP-binding cassette proteins; ANT, aminoglycoside nucleotidyltransferase; APH, aminoglycoside phosphotransferase; Arr, rifampin ADP-ribosyltransferase; BLA, β-lactamase; CAT, chloramphenicol acetyltransferase; DFR, dihydrofolate reductase; Erm, Erm *23S rRNA* methyltransferase; FOS, fosfomycin thiol transferase; LNU, lincosamide nucleotidyltransferase; MATE, multidrug and toxic compound extrusion efflux pump; MFS, major facilitator superfamily efflux pump; SAT, streptothricin acetyltransferase; TRRPP, tetracycline-resistant ribosomal protection protein; Van, glycopeptide resistance gene cluster; VAT, streptogramin acetyltransferase.

Genomic analysis of 1114 WGS revealed a total of 2198 intrinsic and acquired ARGs in the genome sequences of 510 (45.8%) strains (Supplementary Table S3). Overall, we identified 425 acquired ARGs (57 different) in 218 (19.6%) taxonomically diverse strains. As indicated by the phenotypic data, the human intestinal group most frequently carried acquired ARGs (34.5%), which is reflected in their substantial diversity (Table 1, Figure 2a). Although limited genomic data were available for isolates from human milk, acquired ARGs were quite widespread among these strains (26.1%). However, compared to human intestinal isolates, a lower proportion of commercial strains (13.8%, p < .001) and isolates of the natural microbiota from fermented products (referred to as nonstarter strains; 8.7%, p < .001) harbored acquired ARGs (Figure 2b). In starter strains, these genes were rarely present (3.2%) (Table 1).

**Table 1.** Acquired resistance genes (ARGs) and mobile genetic elements (MGEs) detected in strains of four groups of lactic acid bacteria and bifidobacteria.

	ARGs			
	No. of all ARGs	No. of diverse ARGs	No. of strains [%]	No. of species
Human intestinal mucosa or feces	271	44	124 [34.5]	16
Human milk or colostrum	11	5	6 [26.1]	3
Nonstarter strains^a^	54	22	22 [8.7]	10
SPPF^b^	89	24	66 [13.8]	18
Probiotic strains	73	18	55 [18.2]	13
Starter cultures	5	4	5 [3.2]	4
Feed additives	10	6	5 [31.3]	4
Protective cultures	1	1	1 [16.7]	1
	MGEs			
	No. of ARGs associated with MGEs	No. of diverse MGEs	No. of strains [%]	No. of species
Human intestinal mucosa or feces	203	49	79 [22.0]	15
Human milk or colostrum	9	5	6 [26.1]	3
Nonstarter strains^a^	39	14	10 [4.0]	6
SPPF^b^	70	30	54 [11.3]	14
Probiotic strains	59	21	47 [15.6]	8
Starter cultures	2	2	2 [1.3]	2
Feed additives	9	7	5 [31.3]	4
**MGE group**				
Not determined	108	21	47	12
Plasmids	87	24	36	10
Integrative and conjugative elements	57	9	39	9
Insertion sequences	43	6	42	7
Integrative and mobilizable elements (IME)	11	6	11	8
IME-like	9	7	9	7
Composite transposons	3	2	3	3
Prophages/PICIs^c^	3	2	2	2

^a^Isolates of natural microbiota from fermented products

^b^Strains intentionally introduced into the agro-food chain via starter, protective, and probiotic cultures and feed additives

^c^Phage-inducible chromosomal island

Statistical analysis confirmed a significant difference in the mean number of acquired ARGs between four groups of isolates. Commercial (p < .001) and nonstarter (p < .001) strains on average contained fewer acquired ARGs than human intestinal isolates (Figure 2c). Similarly, starter cultures harbored significantly fewer acquired ARGs than probiotic strains (p = .004) (Table 1). We also observed significant differences between the 28 genus pairs, with bifidobacteria and enterococci representing the genera enriched in acquired ARGs (Supplementary Table S2).

**Figure 3.**
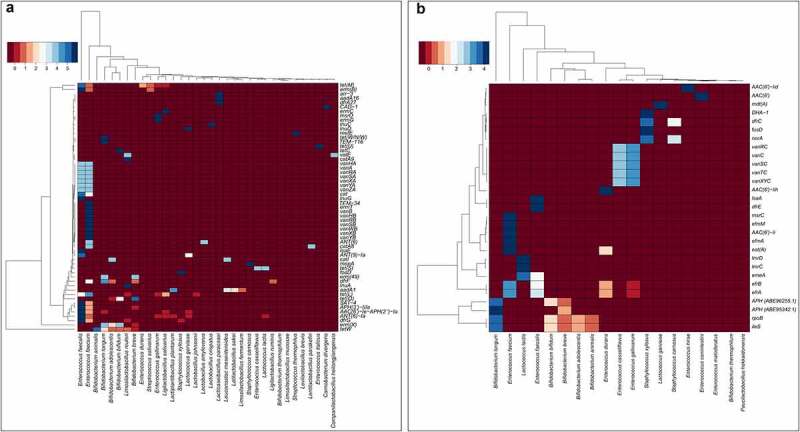
Hierarchical clustering of (a) acquired and (b) intrinsic antimicrobial resistance genes detected in the whole genome sequences of the studied species of lactic acid bacteria and bifidobacteria. Standardized values (Z-score) are shown. Heatmaps were created using heatmap3 (version 3.4.4, The R Foundation, Austria).

Sequencing data showed that the *tetW* gene, encoding tetracycline-resistant ribosomal protection protein (TRRPP), was the most abundant in our samples, as it was detected in 92 (8.3%) strains. This gene was mostly carried by intestinal or probiotic bifidobacteria or lactobacilli. Among others, *erm(X)* (42 strains), *tet(M)* (40), *erm(B)* (29), *ANT(6)-Ia* (21), *AAC(6’)-Ie-APH(2”)-Ia* (16), *APH(3’)-IIIa* (15), *SAT-4* (14), *tet(O)* (13), *tet(L)* (13), and *dfrG* (11) were frequently detected, especially in the genomes of human intestinal isolates. Sequences encoding *erm(X)* and *tet(O)* were typical of bifidobacteria, whereas others were mainly associated with enterococci (Figure 3a). In line with these results, TRRPPs were the most represented class of acquired ARGs in our samples, followed by Erm *23S rRNA* methyltransferases and aminoglycoside nucleotidyltransferases (Figure 2d). Acquired ARGs were rare in *Leuconostoc* sp. and *Lactococcus lactis*, as only 4% (*aadA1, catI*) and 3% (*ANT(6)-Ia, tet(S)*) of whole genome sequences (WGS) contained ARGs, respectively. As expected, no acquired ARGs were identified in pediococci (Figure 3a).

In addition to *tetW*, which accounted for half of the acquired ARGs in commercial strains, *lnuA, tet(L), tet(M)*, or *erm(B)* occurred quite often. Only *dfrF* was present in all sample sources, whereas more than half genes (n = 31) were present in only one particular group (Figure 2a). MDR strains encoding acquired ARGs conferring resistance to three or more groups of antimicrobials were observed at low frequency (10.6% of strains, Figure 2e), predominantly in human intestinal isolates.

The results revealed that non-risky intrinsic ARGs^15^ are indeed a common genomic feature in enterococci, bifidobacteria, and lactococci (Figure 3b). We detected a total of 1773 intrinsic ARGs (29 different) in 462 bacterial strains from different sources. Interestingly, a large proportion of the intrinsic ARGs (43.6%) were efflux pumps (e.g. EfrAB, LmrCD, EmeA, EfmA). Since some strains of enterococci cause severe infections in humans,^16^ antimicrobial resistance of these strains has received much attention in recent years, leading to a more thorough characterization of resistance mechanisms. As a result, several intrinsic ARGs were found in enterococci (Figure 3b), which have been associated with resistance to aminoglycosides (AAC(6’), EfmM), lincosamides, streptogramin A, and pleuromutilin (Lsa(A), Eat(A)), or macrolides and streptogramin B (MsrC). In addition, *E. casseliflavus* and *E. gallinarum* strains encode a *vanC* gene cluster that has been reported to confer low level of vancomycin resistance.^17^ We also discovered that intrinsic aminoglycoside phosphotransferases are typical for bifidobacteria, and an efflux pump LmrCD for *Lactococcus lactis* (Figure 3b).

### Mobile genetic elements

The genetic environment of ARGs was systematically investigated for the presence of MGEs. Our analysis highlights the absence of genomic islands associated with intrinsic ARGs, although individual MGE genes (e.g. an insertion sequence or an integrase) were detected in the genetic environment of some intrinsic ARGs, particularly chromosomal genes that do not confer resistance when not mutated (e.g. *ileS, rpoB, eat(A), dfrE, msrC*).

**Figure 4.**
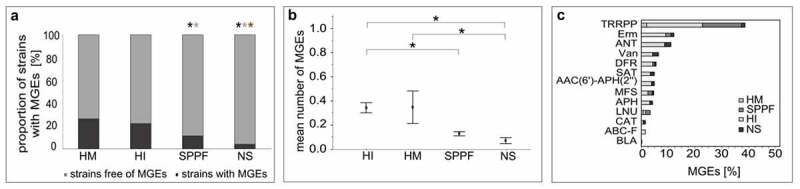
Mobile genetic elements (MGEs) in lactic acid bacteria and bifidobacteria of different origin. (a) The proportion of strains with MGEs. Statistics: Likelihood ratio test, Fisher’s exact probability test; p < .05; black asterisk, statistically significant difference compared to the human intestinal mucosa or feces group (HI); gray asterisk, statistically significant difference compared to the human milk or colostrum group (HM); brown asterisk, statistically significant difference compared to strains intentionally introduced into the agro-food chain (SPPF group). (b) The mean number (± standard error) of MGEs. Statistics: rank analysis of variance (Kruskall-Wallis), pairwise multiple comparisons using Dunn’s test with Bonferroni adjustment; p < .05; *, statistically significant difference. (c) The prevalence of the protein families of ARGs detected in mobile genetic elements (MGEs). NS, isolates of the natural microbiota from fermented products (nonstarter strains).

The comprehensive survey led to the identification of a total of 211 MGEs in 149 strains (68.3% of strains with acquired ARGs), which accounted for a large proportion of the identified acquired ARGs (n = 321, 75.5%) (Supplementary Table S4). Additional BLAST analyses allowed us to distinguish these elements into 77 unique MGEs, of which we considered 34 to be novel. The results of the statistical analyses were consistent with the data on acquired ARGs – a lower proportion of commercial strains (11.3%) and nonstarter strains (4.0%) contained MGEs compared with human isolates (Figure 4(a-b)). In addition, MGEs were found more frequently in probiotics (15.6%) than in starter cultures (1.3%) (Table 1). We also found significant differences among the 14 genus pairs, reflecting the high abundance of MGEs in bifidobacteria and enterococci (Supplementary Table S2).

Our results imply that ARGs in LAB and bifidobacteria are more likely to spread by conjugation rather than transduction. Plasmids, integrative and conjugative elements (ICEs), and insertion sequences were abundant in our strain set, whereas other MGEs were detected less frequently (Table 1). Nevertheless, a considerable number of MGEs could not be classified into a specific group due to the absence of signature genes, possibly due to sequencing or assembly errors. The prevalence of the *tetW, tet(M), ANT(6)-Ia*, and *erm(B)* genes in the WGS of the analyzed strains was reflected in the number of MGEs associated with these ARGs (Figure 4c). The majority of strains possessed one (74.5%) or two (16.1%) MGEs.

**Figure 5.**
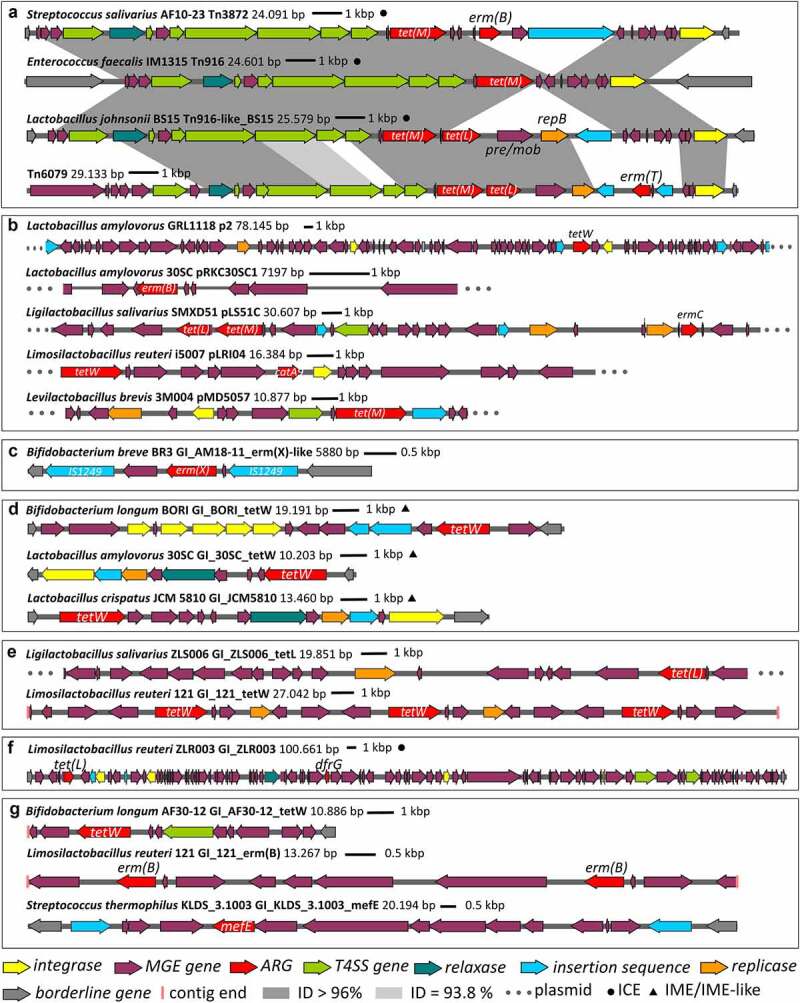
Genetic organization of selected known and putative novel mobile genetic elements (MGEs) found in strains deliberately added into the agro-food chain. Among known MGEs, (a) Tn916 and similar elements, (b) plasmids, and (c) a composite transposon were found. Novel MGEs included (d) integrative and mobilizable (or similar) elements (IME/IME-like), (e) plasmids, (f) integrative and conjugative elements (ICEs), and (g) unclassified MGEs. The labels of novel MGEs were assigned in-house. Genetic organization was visualized using the snapgene-viewer. ARG, antimicrobial resistance gene; ID, BLAST identity; T4SS, type IV secretion system.

Known MGEs mainly included plasmids (e.g. pHTβ-like, pELF1, pV24-1, and pE1_29 in enterococci, pLR585, pLR581, and pLRI04 in *Limosilactobacillus reuteri*, pLS51C in *Ligilactobacillus salivarius*, pMD5057 in *Levilactobacillus brevis*, and pldC in *Lactiplantibacillus plantarum*), but less frequently ICEs (ICE_COH1_guaA, Tn*5801*, Tn*3872*, Tn*1549*-like), and integrative and mobilizable elements (IMEs) or similar elements (ICESsu(SC84)-like, Tn*6260*, ATE-1-like). We observed a high prevalence of pWZ909-like elements^18^ encoding different ARGs (Supplementary Figure S2) in intestinal *E. faecalis* strains. The mobility of *tet(M), tet(L), SAT-4*, and/or *ANT(6)-Ia* in enterococci appears to be linked to Tn*916* or similar elements, as shown in Figure 5a. On the other hand, intestinal and probiotic isolates of bifidobacteria carried *tet(O)* on an element showing nucleotide similarity with a short segment of ICESsuLP081102, as described in our previous study.^9^ In total, 12 different known MGEs were detected in commercial strains, including ICEs (e.g. Tn*916*), plasmids (e.g. p2, pRKC30SC1, pLS51C, pLRI04, pMD5057, Figure 5b), and composite transposons (e.g. *erm(X)*, IS1249, Figure 5c).

In present study, we successfully discovered 17 putative novel MGEs in commercial strains, associated with 10 ARGs (*tetW, tet(L), dfrG, mefE, erm(B), lnuC, dfrF, ANT(6)-Ia, AAC(6’)-Ie-APH(2”)-Ia*, and *erm(49)*). These elements were classified as IME or similar elements (Figure 5d), plasmids (Figure 5e), insertion sequences, ICEs (figure 5f), prophages, composite transposons, or other unclassified MGEs (Figure 5g). Known MGEs frequently carried *TRRPP* (48.2%), *DHFR* (14.3%), and *erm* (10.7%) genes.

### Pan- and core-genome analysis

By analyzing pan-genomes, we identified core and accessory genes of 36 species. Despite the fact that strains of the subspecies *B. animalis* subsp. *lactis* are known to encode *tetW*,^19^ it is one of the most commonly used species in dietary supplements. A genomic island containing *tetW* and an adjacent transposase was confirmed in the majority of *B. animalis* subsp. *lactis* strains.^20^ The pan-genomes of the 38 *B. animalis* subsp. *lactis* and eight *B. animalis* subsp. *animalis* isolates comprised 3239 orthologous ORFs, of which 834 represented the core genome (Supplementary Figure S3A). Surprisingly, *B. animalis* subsp. *lactis* strains containing *tetW* showed close genomic relatedness as they clustered into a monophyletic clade. Notably, three *B. animalis* subsp. *lactis* isolates of animal origin were devoid of *tetW* (2010B, 2007B, and 2011B) and clustered into two separate branches, suggesting greater genomic distance. The results suggest that the genomic island was integrated into the common ancestor before the strain ATCC 27673 isolated from sewage diverged. This indicates a limited number of closely related strains circulating in the industry, but also the lack of sampling of this subspecies from other environments.

The phylogeny of the 53 *E. faecium* strains confirmed two major clades, A and B (Supplementary Figure S3B). As expected, the commensal strain clustered into clade B, whereas the animal and pathogenic isolates clustered into clade A.^16^ The epidemic hospital strains clustered into a subclade that was genetically more distant from other clade strains of clade A, mainly nonstarter isolates. Interestingly, the ARGs were enriched in a subclade with pathogenic isolates. Commercial strains were generally not found in a subclade with pathogenic strains and did not contain ARGs.

### Metagenome sequences analysis

The transmission potential of MGEs was examined by screening contigs of metagenomic sequences, mostly those of the human fecal microbiota. We provide evidence of substantial horizontal spread of several MGEs found in whole genome sequences of LAB and bifidobacteria (Supplementary Table S5).

Specifically, 24 of 77 different MGEs were detected in 883 (50.2%) metagenomic samples, seven of which were from commercial strains (Table 2). The results suggest that several of these (mostly short) elements carrying *lnuC, tet(O), erm(B)*, or *tet(M)* have high transmission potential in the human gut microbiota, as they were detected in a number of samples on contigs with high estimated taxonomic diversity. On the other hand, the findings indicate that the genomic islands of the probiotic strains *B. longum* IM810 (*erm(49)*) and *B. animalis* subsp. *lactis* strains (*tetW*) were associated only with the (sub)species of origin despite their presence in several metagenomic samples. Similarly, the resistance plasmids of *Limosilactobacillus reuteri* (pLR581 in pLR585) were not detected outside the species.

**Table 2.** The prevalence of mobile genetic elements (MGEs) found in commercial strains among the analyzed metagenomic samples.

ARG	MGE^a^	No. of samples	No. of species	Metagenome
*lnuC*	GI_C25_lnuC	508	66	HF
*tet(O)*	ICESsuLP081102-like	217	36	HF
*erm(B)*	GI_BS15_erm(B)	104	25	HF, HMM
*tet(M)*	Tn916	88	29	HF, HMM
*erm(49)*	GI_IM810	32	1	HF
*tetW*	ISBian1 GI	7	1	HF
*erm(X)*	GI_AM18-11_erm(X)-like	5	3	HF

ARG, antimicrobial resistance gene; HF, metagenomic sequences of human fecal microbiota, HMM, metagenomic sequences of human milk microbiota.

^a^Labels of novel MGEs were assigned in-house. See Supplementary Table S4.

The results showed that ICEs and IMEs were less prevalent in the human fecal microbiota. In addition to the widely disseminated Tn*916*, Tn*5801* was also detected. Novel IMEs carrying *dfrF* were found in multiple samples, wherease others harboring *tetW, ANT(6*), and *lnuG* (e.g. Tn*6260*, ATE-1-like) occurred sporadically. Surprisingly, plasmids were rarely detected (e.g. pRE25-like). The described findings were confirmed by online WGS analysis of the RefSeq Genome Database (bacteria, taxid:2), where even more diverse and longer MGEs were detected in bacteria from various sources (Supplementary Table S5). Metagenomic samples of the human milk microbiota and the microbiota of fermented products were not burdened with ARGs and MGEs.

## Discussion

Starter, probiotic, and protective cultures or feed additives consumed with commercial products come into contact with a dense microbial community in the intestinal environment, where they may allow for ARGs dissemination.^21^ Given that we do not yet know the actual size of the resistance gene pool in commercial strains or how rapidly they spread through the host microbiota, information on the associated safety risk is limited. In this context, our aim was not only to determine phenotypic susceptibility but also to perform a comprehensive and comparative screening for ARGs and MGEs in the genomes of four groups of LAB and bifidobacteria. We evaluated the significance of the discovered elements by examining metagenomic sequences.

Phenotypic susceptibility data showed a moderate overall resistance rate (14.6%). Since epidemiological cutoff values are defined for a limited number of bacterial species,^3^ our extensive susceptibility data will be useful for their revision. Resistance was less prevalent in commercial strains than in intestinal strains (Figure 1), while starter cultures were generally more susceptible than probiotics. Studies have reported phenotypic resistance in strains from human milk,^22^ feces,^5,23^ fermented products,^24^ probiotics, feed additives, and starter cultures,^25–28^ yet a comparative analysis between the four groups has not been performed.

Resistance occurred significantly more frequently in *Levilactobacillus* and *Pediococcus*, while *Streptococcus thermophilus* displayed pan-susceptibility, which is in contrast to other studies that reported streptomycin, erythromycin, kanamycin, and tetracycline resistance.^29,30^ Since the distributions of MICs in pediococci were unimodal, which is typical of intrinsic resistance, the cutoff values in the guidelines^3^ appear to be too low. We propose to increase the cutoff values for kanamycin, neomycin, tetracycline, chloramphenicol, and ampicillin in pediococci (Supplementary Figure S1), which was also suggested by Shani et al. (2021) for chloramphenicol and tetracycline.^31^

A comprehensive genome-wide comparative analysis based on the 1114 genomes led to the identification of intrinsic and acquired ARGs and associated MGEs. Acquired ARGs raise safety concerns when present in commercial strains.^3^ Results showed that one-fifth of the strains carried acquired ARGs, with those involved in resistance to tetracyclines and macrolides being the most common (Figure 2d). This reflects their intensive use today or in the past.^32^ The genes *tet(M), erm(B), ANT(6)-Ia, AAC(6’)-Ie-APH(2”)-Ia, APH(3’)-IIIa, SAT-4, tet(L)*, and *dfrG* (Figure 3a) were prevalent in our strain set, mainly in enterococci, but were also found in bifidobacteria and lactobacilli, consistent with other reports.^4,6,33–36^ We also confirmed that *erm(X)* and *tet(O)* are typical among bifidobacteria.^34^

Horizontal transfer of ARGs is driven by mobile genetic elements. Despite their importance for safety risk assessment, these elements have not yet been systematically studied in many genera of LAB and bifidobacteria. The majority of acquired ARGs (75.5%) were found within MGEs (e.g. plasmids, ICEs, and insertion sequences) and most commonly encoded resistance to tetracycline, macrolides, and aminoglycosides. Jiang et al. (2019) likewise reported that ARGs are spread by conjugation rather than transduction.^37^ In addition to the known elements, we also discovered 34 that had to our knowledge not been previously reported in the literature (Figure 5d-5g). Due to the many long repetitive elements common in MGEs, copy number alterations, and structural variations in bacterial genomes, short-read sequencing technologies can result in gaps and shorter assembled contiguous sequences.^38^ This can affect our ability to annotate the complete MGEs, which can span through multiple contigs. The use of long-sequencing technologies can help overcome this issue by allowing resolution of these repetitive elements and removing ambiguities in their position or size.^38^ Intrinsic ARGs were frequently detected in our strains (Figure 3b), but are not considered risky due to the absence of MGEs,^39^ which our results confirmed.

In agreement with the analysis of acquired resistance genes, MGEs were most abundant in bifidobacteria and enterococci. Foodborne enterococci are rarely implicated as pathogens,^6^ but may serve as vehicles for resistance transmission,^10^ which was confirmed in present study. In contrast to our results, Mancino et al. (2019) found that mobile ARGs accounted for only a small fraction of the total resistome (1.5%) of bifidobacteria.^40^ MGEs were also not prevalent in other genera of LAB, however, these bacteria may carry plasmids,^8^ IMEs, or ICEs.^41–43^

Estimating the real risk of ARGs harbored by environmental bacteria is not an easy task and should be based on the evaluation of the likelihood that these genes will be introduced into pathogenic bacteria and ultimately compromise the efficacy of antibiotic therapy. Factors that influence the risk of a particular ARG in a commensal strain include its association with a MGE, substrate specificity, fitness cost, and ecological niche overlap with human pathogens.^39^ Given the lower abundance and diversity of ARGs (and MGEs) coupled with similar substrate profiles in bacteria deliberately introduced into the agro-food chain compared to human intestinal isolates (Figures 2 and 4, Table 1), we estimate that this group generally does not pose a high risk. This is consistent with the requirement for absence of acquired ARGs in commercial strains.^2^ The higher prevalence of resistance in human gut isolates may be attributed to the fact that this environment is more frequently exposed to antibiotics and selection pressure.^44,45^ To date, only a handful of studies have been conducted on antimicrobial resistance in commercial strains based on WGS analyses, which revealed a low prevalence of acquired ARGs.^7,8,20^

Most people consume larger amounts of starter cultures or nonstarter bacteria other than probiotics, in addition to fermented products. Based on the results obtained, we do not consider these two groups as a risk due to the low incidence of ARGs and MGEs (Table 1). These bacteria come from environments^46^ that are not as frequently exposed to antimicrobials. Probiotics, on the other hand, are selected to survive intestinal passage and usually originate from the human gut,^47^ so they are more likely to contain risky elements than starter cultures. Such individual commercial strains harboring acquired ARGs may indeed pose a risk.

High-throughput metagenomic analysis provides a powerful alternative to laboratory analyses of MGE mobility and is an effective tool for evaluating the transmission potential of MGEs. We have gained important insight into the prevalence of MGEs (Supplementary Table S5), which substantially aids the risk analysis. To our knowledge, such studies have not yet been performed in this group of bacteria. The fact that these were mostly short genomic islands, IMEs, or ICEs, but rarely plasmids, may be related to sequencing or assembly errors, which had also been reported previously.^48^

We have demonstrated that the elements carrying *lnuC, erm(B), tet(O)*, and *tet(M)* have the potential for transmission within the gut microbiota. The widespread distribution of the Tn*916* carrying *tet(M)* in the human gut has been reported previously.^49^ Conversely, no evidence of mobility outside the host species (*B. longum* or *B. animalis* subsp. *lactis*) was found for elements bearing *erm(49)* and *tetW*, which is consistent with reports that *erm(49)* does not occur in the human fecal microbiota^50^ and that *tetW* from *B. animalis* subsp. *lactis* is not transferable to other species *in vitro*.^51^

Because the microbiota of fermented foods was not burdened with MGEs, the risk of these products is negligible, which is consistent with the findings of Walsh et al. (2020).^52^ Contrary to expectations from prior studies, human milk samples were not burdened with MGEs.^53^ However, less sequence data were analyzed for human milk samples because they are heavily contaminated with host DNA and have low microbial abundance.^54^

The results of present study expand our knowledge of transferable resistance in strains deliberately introduced into the agro-food chain and provide a basis for risk assessment analyses that is currently lacking. To the best of our knowledge, this was the first study of this group of bacteria on such a large scale, as advanced approaches are slower to be implemented in commensal bacteria than in medically important bacteria. Considering that transferable resistance was more common in strains of human origin, commercial strains do not significantly increase the ARG gene pool and thus do not pose a serious threat to human health. Nevertheless, special attention should be paid to individual commercial strains (mostly probiotics) that contain elements that have been shown to have a high potential for transferability in the gut microbiota.

## Material and methods

### Isolation of bacterial strains and propagation

Four groups of LAB and bifidobacteria were included in the analyses: (1.) 157 strains from starter and protective cultures, dietary supplements (probiotics), and feed additives (referred to as commercial strains), (2.) 154 isolates of the natural microbiota from fermented products (referred to as nonstarter strains), (3.) 90 isolates from human intestinal mucosa or feces, and (4.) 73 isolates from human milk or colostrum. Primary dilutions of fermented dairy samples, dietary supplements and commercial cultures (Supplementary Table S1) were prepared in sodium citrate solution (concentration 0.02 g/ml, pH = 7.5) (Sigma-Aldrich, W302600-1 KG-K) or buffered peptone water (Merck, 1.07228.0500), respectively. Serial 10-fold dilutions (¼ Ringer solution, Merck, 1.15525.0001) of the homogenates were plated (100 μl) onto the selective agar media: Rogosa, MRS, M17 (Merck, 1.05413.0500, 1.10661.0500, 1.15108.0500), and/or TOS-MUP (Yakult Honsha, 8-MJ54). After incubation in appropriate conditions (Supplementary Table S6), distinct colonies were picked for Random Amplified Polymorphic DNA (RAPD) analysis using the M13 primer (5’-GAGGGTGGCGGTTCT-3’) as described previously.^55^ Colonies were subcultured on the appropriate media to obtain pure cultures and stored at −80 °C. A few probiotic and starter strains were obtained from the manufacturer or from the culture collection (Supplementary Table S1). Bacterial strains from human biological samples were isolated previously^56,57^ and stored in the culture collection of the Institute of Dairy Science and Probiotics (Biotechnical faculty, University of Ljubljana, Slovenia) and ZIM culture collection (https://www.zim-collection.si/), which is a member of the World Federation of Culture Collections (#810). Strains were subcultured (1% v/v) at least twice before the experiments (Supplementary Table S6).

### Isolation of genomic DNA and bacterial species identification

Bacterial cells (1 ml of overnight culture) were collected by centrifugation (3 min, 12 000 g) (Hettich, Germany) and incubated in 500 µl of TE buffer containing mutanolysin (25 U/ml) and lysozyme (10 mg/ml) for 2 hours at 37°C. Genomic DNA was extracted using the ISOLATE II Genomic DNA Kit (Bioline, BIO-52067) or the Wizard® Genomic DNA Purification Kit (Promega, A1120) according to the manufacturer’s instructions.

Isolated strains were identified at the species level by (1.) PCR, according to previously developed protocols, and species-specific primers (see Supplementary Table S7), (2.) matrix-assisted laser desorption/ionization time-of-flight (MALDI-TOF) mass spectrometry (Microflex LT system; Bruker Daltonics, Germany), or (3.) sequencing of the 16S rDNA genes (Microsynth, Switzerland) (Supplementary Table S7). Sequencing was done at Microsynth AG (Switzerland) and the 16S rDNA sequences were analyzed using the BLAST algorithm.^58^

### Antimicrobial susceptibility testing

The broth microdilution method and precoated microtiter plates VetMIC Lact-1 (Statens Veterinärmedicinska Anstalt, Sweden) were used to determine the MICs of clinically relevant antimicrobials (gentamicin, kanamycin, streptomycin, neomycin, tetracycline, erythromycin, clindamycin, chloramphenicol, ampicillin, vancomycin, tylosin)^3^ for 371 strains according to the standard ISO 10932.^59^ Tylosin, vancomycin, and ampicillin plates were prepared manually. MICs were read visually after plates were incubated anaerobically using the GenBox system (BioMerieux, Marcy l’Etoile, France) for 48 h (bifidobacteria for 72 h) at temperatures indicated in Supplementary Table S6. Enterococci and staphylococci were incubated for 24 h under aerobic conditions. Strains were classified as resistant or susceptible according to the defined epidemiological cutoff values (ECOFFs) .^3^ The quality of phenotypic susceptibility testing was controlled using the reference strains *Lacticaseibacillus paracasei* ATCC 334, *Lactiplantibacillus plantarum* ATCC 14917, *Bifidobacterium longum* ATCC 15707, *Lactococcus lactis* ATCC, *Enterococcus faecalis* ATCC 29212, and *E. faecalis* ATCC 51299.

Phenotypic susceptibility patterns of 103 strains of LAB and bifidobacteria obtained in our previous studies^9,20^ were included in the data analyses to perform as comprehensive statistical analyses and safety assessment as possible. Thus, our total strain set consisted of 474 isolates (157 commercial strains, 154 nonstarter strains, 90 isolates from human intestinal mucosa or feces, and 73 from human milk or colostrum). The isolates were assigned to 50 different species and 18 genera (Supplementary Table S1).^9,20^

### Screening for antimicrobial resistance genes and mobile genetic elements

WGS of four groups of LAB and bifidobacteria (n = 1011, Supplementary Table S8) selected from literature searches or metadata available at BioSamples were retrieved from NCBI FTP^60^ in December 2019 and subjected to quality control analysis. Statistical genomic data were obtained using QUAST 5.0.2,^61^ contamination was estimated using Mash Screen 2.0,^62^ and taxonomic affiliation of strains was verified by calculating Mash distance to type strains. WGS screening data of 103 strains from our former studies,^9,20^ of which 75 were sequenced in-house, were included in the statistical analyses. Our target database thus consisted of 1114 genomes representing 479 strains deliberately added into the agro-food chain, 253 nonstarter strains, 23 strains from human milk, and 359 strains from human intestinal mucosa or feces.

Using BLAST 2.10.0+ (parameters -evalue 1e-10, -max_target_seqs 10, query coverage ≥ 60%, similarity cutoff > 70%), genomic data were scanned for ARGs by comparing sequences in the ARGs database as we described previously.^9^ Briefly, the ARGs database consisted of five publicly available databases (CARD 3.0.8,^63^ ResFinder (2020–02-11),^64^ ARG-ANNOT (V6, 2019),^65^ KEGG (November, 2017),^66^ and NCBI’s Bacterial Antimicrobial Resistance Reference Gene Database (March, 2020)^67^). The intrinsic and acquired nature of ARGs was determined by the pan-genome and MGEs analyses. Pan-genome analyses were done using Roary 3.13.0,^68^ which allowed us to determine which ARGs are part of the core genome (BLASTP identity ≥ 95%, ORF present in ≥ 99% of strains) and which are part of accessory genes, suggesting that they may have been horizontally acquired.

Systematic screening for MGEs in the genetic environment of predicted ARGs (15 coding sequences (CDSs) upstream and downstream) was conducted with BLAST (parameters -evalue 1e-10, -max_target_seqs 10, query coverage ≥ 80%, similarity cutoff ≥ 80%) and a custom MGEs database.^9^ Genomic islands (chromosomal regions acquired by horizontal gene transfer) and their insertion sites were traced using multiple genome alignments generated by progressiveMauve.^69^ Additional BLAST alignments were performed to eliminate the redundancy and to uncover known MGEs published in the literature as well as putative novel elements whose labels were assigned in-house. Protein domain analysis was performed using hmmsearch (–noali -E 1e-10)^70^ and the Pfam database 33.1.

Annotation of the MGE genes allowed the classification of elements into groups based on their signature genes. A putative MGE was annotated as an ICE if it encoded an integrase, a relaxase, and a type IV secretion system, whereas an IME did not contain type IV secretion system genes. MGEs containing an integrase or a relaxase were classified as IME-like elements and elements containing a replicase (Rep) as plasmids. MGEs carrying a transposase were annotated as insertion sequences. Two insertion sequences of the same group were typical of a composite transposon. Prophages contained phage gene homologs, while a phage-inducible chromosomal island lacked structural and lytic genes.

### Metagenome sequences analysis

The potential for transmission of the discovered elements was evaluated by investigating metagenomic sequences of the human fecal microbiota, the microbiota of fermented products and human milk. The assembled contigs were retrieved from online public databases: 1759 samples of human feces microbiota and 122 of fermented products microbiota from 30 BioProjects, including metaHIT and Human Microbiome Project (HMP) (Accessions in Supplementary Table S5). Feces from healthy adults or infants as well as from people with different pathologies, various cheeses, kefir grains, kimchi, fermented sausages, traditional Korean fermented soybean paste (doenjang), and kombucha were analyzed. On the other hand, contigs of human milk microbiota were assembled in-house. Raw reads (Accessions in Supplementary Table S5) were retrieved from ENA (EMBL-EBI, United Kingdom). After quality inspection by FastQC 0.11.9 (Babraham Bioinformatics, United Kingdom), raw reads were filtered and trimmed using TrimmomaticPE 0.39,^71^ and human reads were removed by mapping to the human genome (GRCh38) using Bowtie2 2.3.4.1.^72^ Reads were assembled *de novo* using metaSPAdes 3.15.0.^73^

The sequences of MGEs found in the analyzed WGS were retrieved using SAMtools (version 1.7).^74^ Their presence in the metagenomes was screened with a custom script based on BLAST (similarity ≥ 90%, coverage ≥ 80%). Taxonomic classification of contigs containing MGEs was performed using Kraken2 (version 2.0.9-beta).^75^ The prevalence of MGEs among all published bacterial genomes was confirmed by the online BLAST alignments to the RefSeq Genome Database (bacteria, taxid:2).

### Statistical analyses

SPSS v. 28 (IBS, United Kingdom) was used for the statistical analyses. We used Kruskall-Wallis non-parametric rank analysis of variance to detect statistically significant differences in the mean number of phenotypic resistances and the mean number of acquired ARGs and MGEs among the four groups of bacteria or among genera. As a post-hoc test, we used Dunn’s multiple comparison test with Bonferroni adjustment. The likelihood ratio test and Fisher’s exact probability test were used to detect differences in the proportion of phenotypically resistant strains and the proportion of strains carrying ARGs and MGEs among the four groups. P-values < 0.05 were considered statistically significant.

## Supplementary Material

Supplemental Material

## Data Availability

The authors confirm that the data supporting the findings of this study are available within the article and/or its supplementary materials. The whole-genome sequencing data of the original study have been submitted to the European Nucleotide Archive (ENA) under the project accession PRJEB49530. Databases and codes are available in figshare at https://doi.org/10.6084/m9.figshare.c.6063839.v2.
