# Supplementary material for: Lactic acid bacteria and bifidobacteria deliberately introduced into the agro-food chain do not significantly increase the antimicrobial resistance gene pool
Source: Gut Microbes. 2022 Sep 28;14(1):2127438. doi: 10.1080/19490976.2022.2127438 (PMC9542798; doi:10.1080/19490976.2022.2127438)
Supplement: Supplemental Material [file KGMI_A_2127438_SM4927.zip › supplementary.zip!/Supplementary Figure S2.pdf]

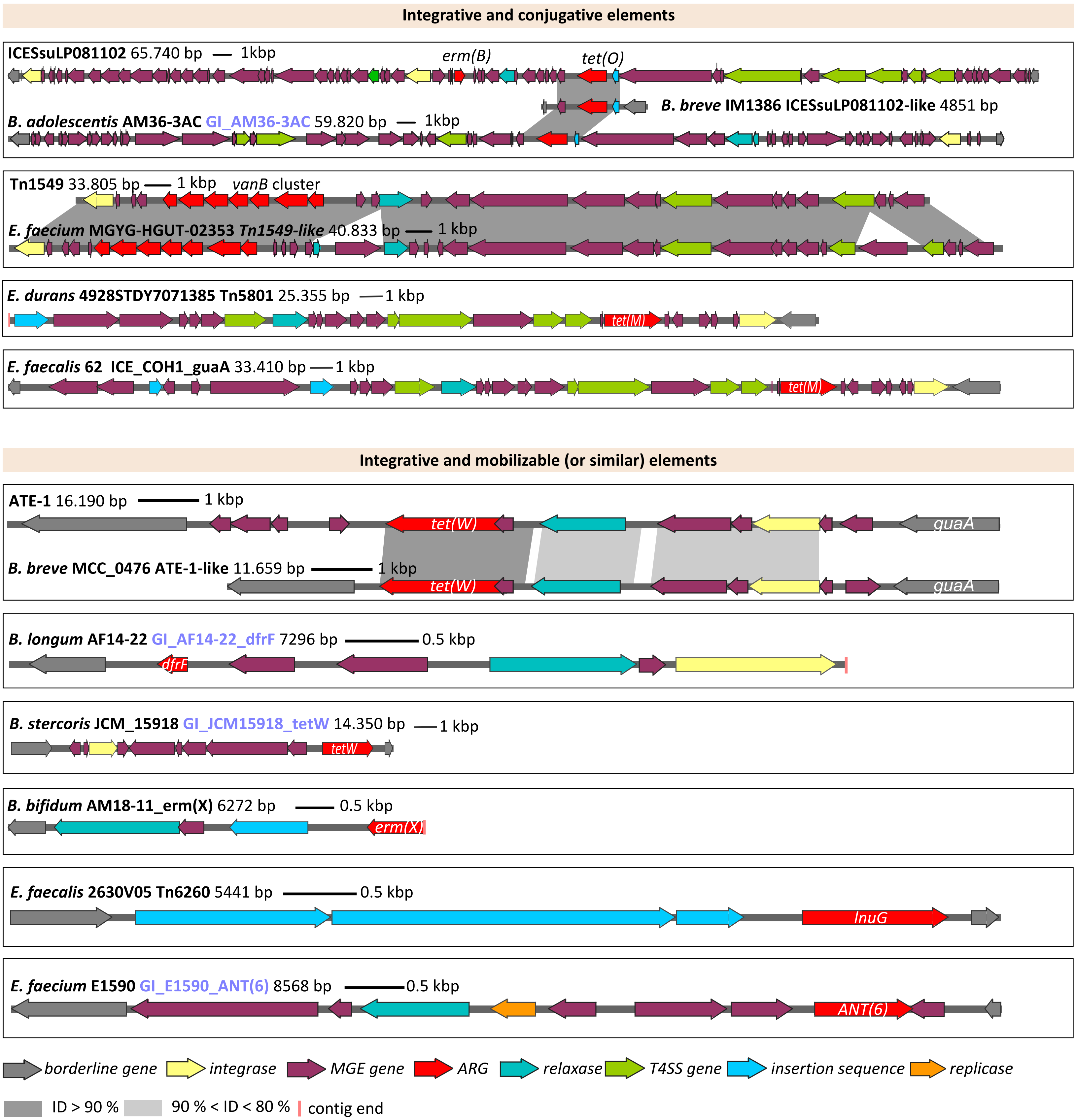
Integrative and conjugative elements
ICESsuLP081102 65.740 bp 1kbp
erm(B)
tet(O)
B. breve IM1386 ICESsuLP081102-like 4851 bp
B. adolescentis AM36-3AC GI_AM36-3AC 59.820 bp 1kbp
Tn1549 33.805 bp 1 kbp vanB cluster
E. faecium MGYG-HGUT-02353 Tn1549-like 40.833 bp 1 kbp
E. durans 4928STDY7071385 Tn5801 25.355 bp 1 kbp
tet(M)
E. faecalis 62 ICE_COH1_guaA 33.410 bp 1 kbp
tet(M)
Integrative and mobilizable (or similar) elements
ATE-1 16.190 bp 1 kbp
tet(W)
guaA
B. breve MCC_0476 ATE-1-like 11.659 bp 1 kbp
tet(W)
guaA
B. longum AF14-22 GI_AF14-22_dfrF 7296 bp 0.5 kbp
dfrF
B. stercoris JCM_15918 GI_JCM15918_tetW 14.350 bp 1 kbp
tetW
B. bifidum AM18-11_erm(X) 6272 bp 0.5 kbp
erm(X)
E. faecalis 2630V05 Tn6260 5441 bp 0.5 kbp
lnuG
E. faecium E1590 GI_E1590_ANT(6) 8568 bp 0.5 kbp
ANT(6)
borderline gene
integrase
MGE gene
ARG
relaxase
T4SS gene
insertion sequence
replicase
ID > 90 %
90 % < ID < 80 %
contig end

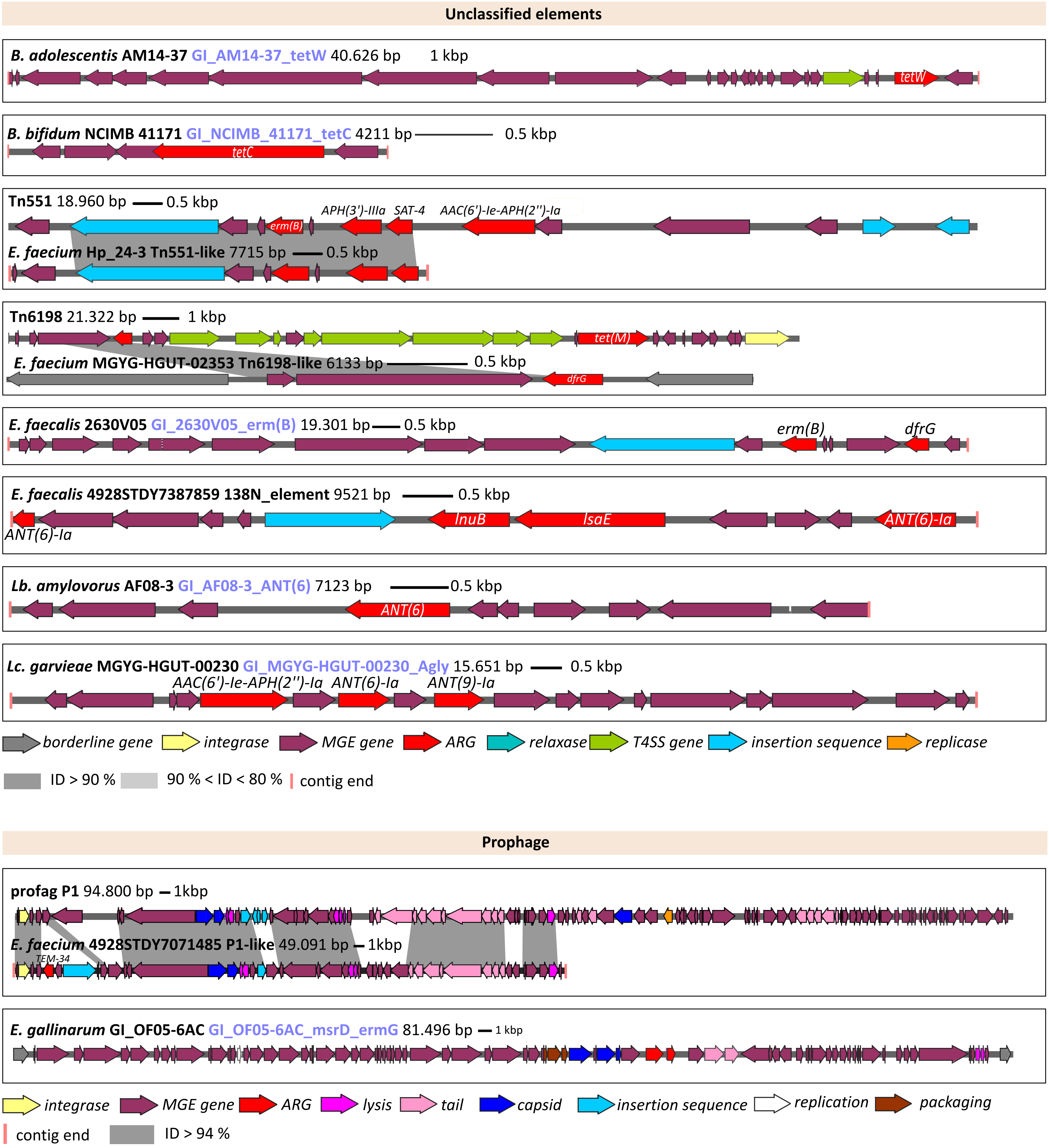
Unclassified elements
B. adolescentis AM14-37 GI_AM14-37_tetW 40.626 bp
1 kbp
tetW
B. bifidum NCIMB 41171 GI_NCIMB_41171_tetC 4211 bp
0.5 kbp
tetC
Tn551 18.960 bp
0.5 kbp
APH(3')-IIIa
SAT-4
AAC(6')-Ie-APH(2'')-Ia
erm(B)
E. faecium Hp_24-3 Tn551-like 7715 bp
0.5 kbp
Tn6198 21.322 bp
1 kbp
tet(M)
E. faecium MGYG-HGUT-02353 Tn6198-like 6133 bp
0.5 kbp
dfrG
E. faecalis 2630V05 GI_2630V05_erm(B) 19.301 bp
0.5 kbp
erm(B)
dfrG
E. faecalis 4928STDY7387859 138N_element 9521 bp
0.5 kbp
lnuB
lsaE
ANT(6)-Ia
ANT(6)-Ia
Lb. amylovorus AF08-3 GI_AF08-3_ANT(6) 7123 bp
0.5 kbp
ANT(6)
Lc. garvieae MGYG-HGUT-00230 GI_MGYG-HGUT-00230_Agly 15.651 bp
0.5 kbp
AAC(6')-Ie-APH(2'')-Ia
ANT(6)-Ia
ANT(9)-Ia
borderline gene
integrase
MGE gene
ARG
relaxase
T4SS gene
insertion sequence
replicase
ID > 90 %
90 % < ID < 80 %
contig end
Prophage
profag P1 94.800 bp
1kbp
E. faecium 4928STDY7071485 P1-like 49.091 bp
1kbp
TEM-34
E. gallinarum GI_OF05-6AC GI_OF05-6AC_msrD_ermG 81.496 bp
1 kbp
integrase
MGE gene
ARG
lysis
tail
capsid
insertion sequence
replication
packaging
contig end
ID > 94 %

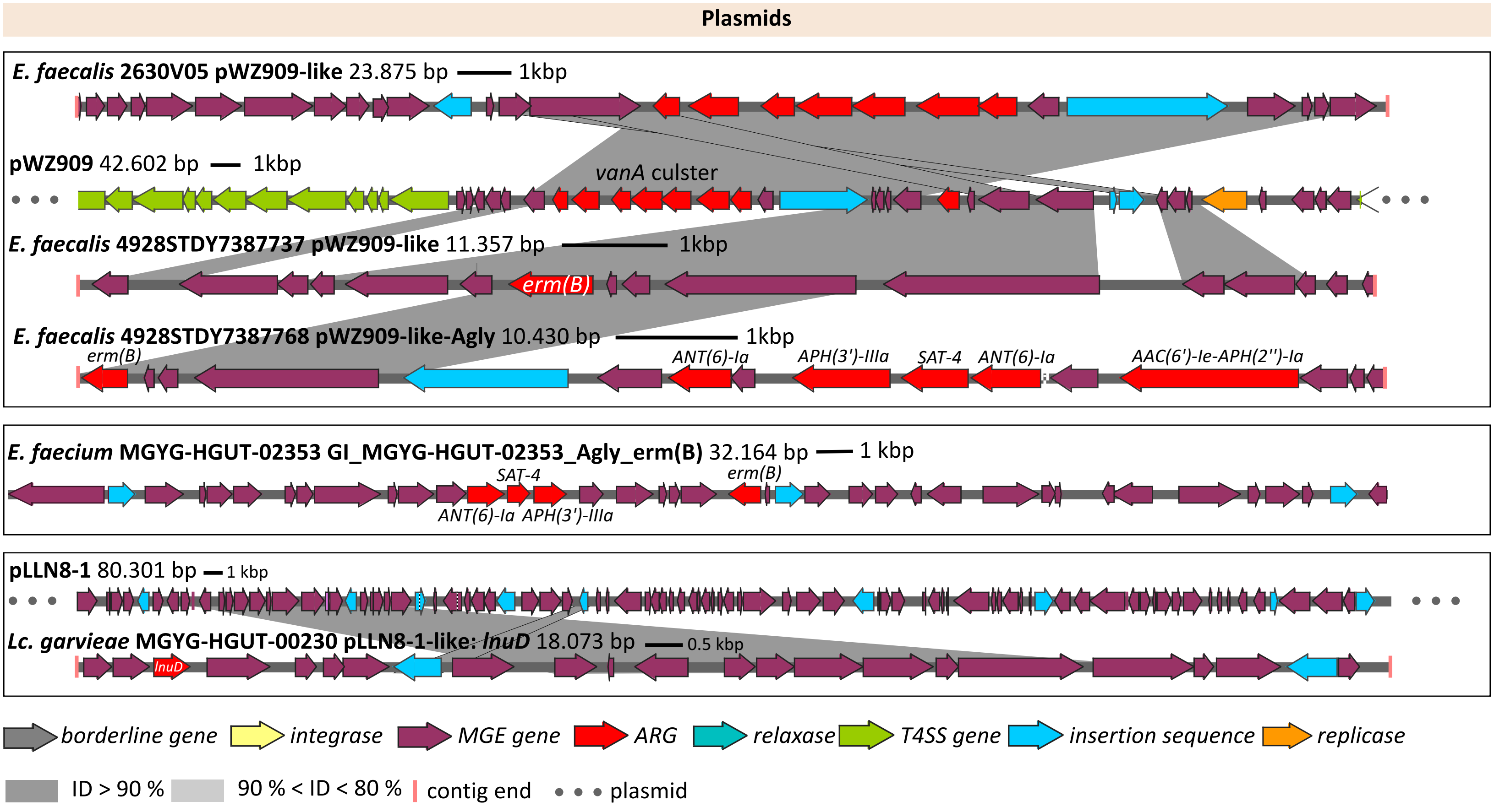

Plasmids
E. faecalis 2630V05 pWZ909-like 23.875 bp
1kbp
pWZ909 42.602 bp
1kbp
vanA culster
E. faecalis 4928STDY7387737 pWZ909-like 11.357 bp
1kbp
erm(B)
E. faecalis 4928STDY7387768 pWZ909-like-Agly 10.430 bp
1kbp
erm(B)
ANT(6)-Ia
APH(3')-IIIa
SAT-4
ANT(6)-Ia
AAC(6')-Ie-APH(2'')-Ia
E. faecium MGYG-HGUT-02353 GI_MGYG-HGUT-02353_Agly_erm(B) 32.164 bp
1 kbp
SAT-4
erm(B)
ANT(6)-Ia
APH(3')-IIIa
pLLN8-1 80.301 bp
1 kbp
Lc. garvieae MGYG-HGUT-00230 pLLN8-1-like: lnuD 18.073 bp
0.5 kbp
lnuD
borderline gene
integrase
MGE gene
ARG
relaxase
T4SS gene
insertion sequence
replicase
ID > 90 %
90 % < ID < 80 %
contig end
plasmid

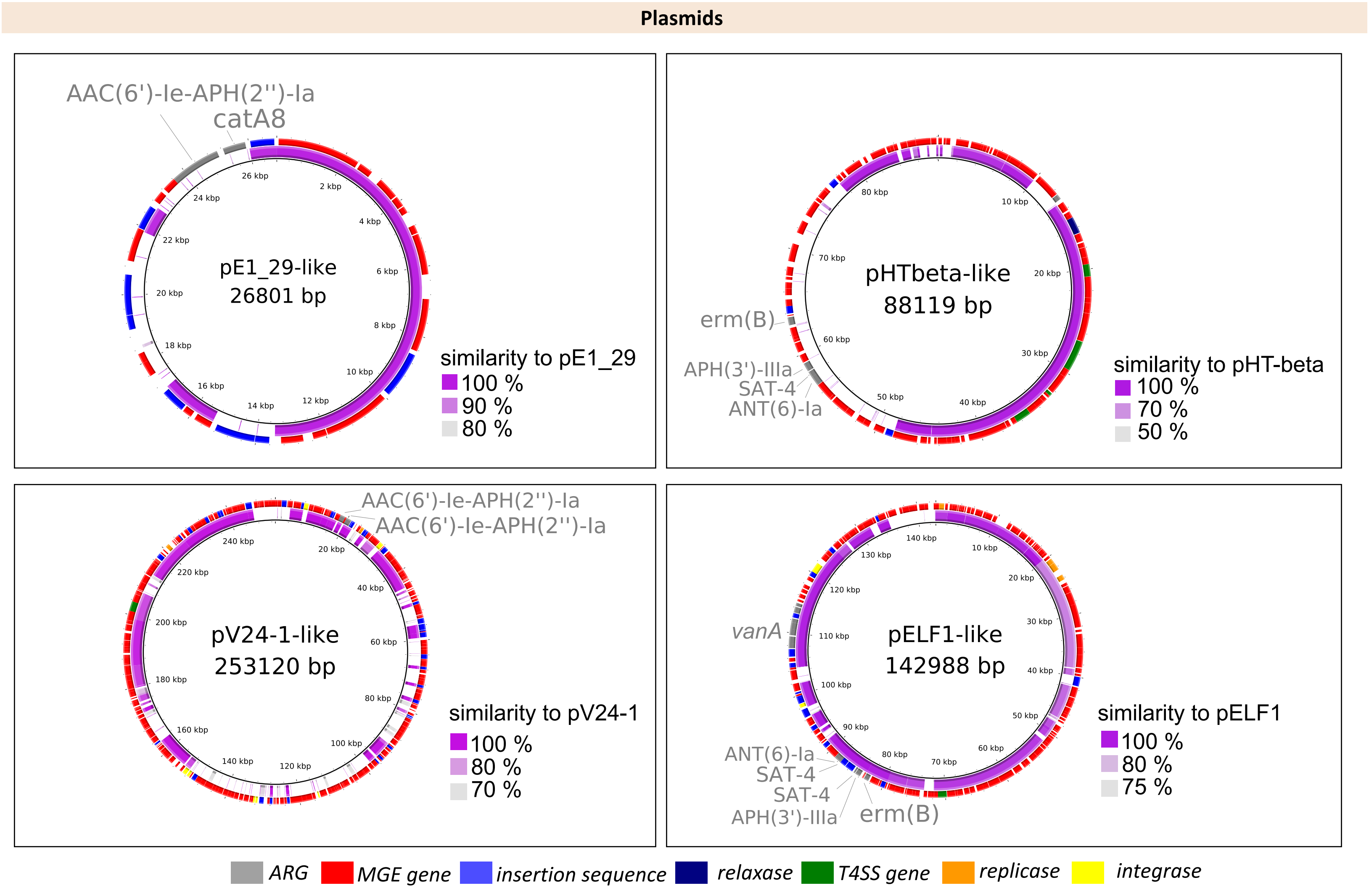

Plasmids
AAC(6')-Ie-APH(2'')-Ia
catA8
pE1_29-like
26801 bp
similarity to pE1_29
100 %
90 %
80 %
pHTbeta-like
88119 bp
erm(B)
APH(3')-IIIa
SAT-4
ANT(6)-Ia
similarity to pHT-beta
100 %
70 %
50 %
AAC(6')-Ie-APH(2'')-Ia
AAC(6')-Ie-APH(2'')-Ia
pV24-1-like
253120 bp
similarity to pV24-1
100 %
80 %
70 %
vanA
pELF1-like
142988 bp
ANT(6)-Ia
SAT-4
SAT-4
APH(3')-IIIa
erm(B)
similarity to pELF1
100 %
80 %
75 %
ARG
MGE gene
insertion sequence
relaxase
T4SS gene
replicase
integrase
