# Supplementary material for: Lactic acid bacteria and bifidobacteria deliberately introduced into the agro-food chain do not significantly increase the antimicrobial resistance gene pool
Source: Gut Microbes. 2022 Sep 28;14(1):2127438. doi: 10.1080/19490976.2022.2127438 (PMC9542798; doi:10.1080/19490976.2022.2127438)
Supplement: Supplemental Material [file KGMI_A_2127438_SM4927.zip › supplementary.zip!/Supplementary Table S2.docx]

Supplementary Table S2. Statistically significant differences in (A) the mean number of minimum inhibitory concentrations exceeded, (B) of mobile genetic elements, and (C) of acquired antimicrobial resistance genes among bacterial genera.

|  | **Genera** | **p** |  | **Genera** | **p** |
| --- | --- | --- | --- | --- | --- |
| (A.) **Phenotypic resistance** | *Streptococcus-Leuconostoc* | 0.048 | **(B.) No. of MGEs** | *Lactococcus-Bifidobacterium* | 0.000 |
|  | *Streptococcus-Lactiplantibacillus* | 0.004 |  | *Lactococcus-Enterococcus* | 0.000 |
|  | *Streptococcus-Levilactobacillus* | 0.000 |  | *Levilactobacillus-Enterococcus* | 0.032 |
|  | *Streptococcus-Bifidobacterium* | 0.000 |  | *Lactobacillus-Bifidobacterium* | 0.000 |
|  | *Streptococcus-Enterococcus* | 0.006 |  | *Lactobacillus-Enterococcus* | 0.000 |
|  | *Streptococcus-Limosilactobacillus* | 0.010 | **(C.) No. of ARGs** | *Pediococcus-Bifidobacterium* | 0.001 |
|  | *Streptococcus-Pediococcus* | 0.000 |  | *Loigolactobacillus-Staphylococcus* | 0.032 |
|  | *Staphylococcus-Levilactobacillus* | 0.001 |  | *Pediococcus-Staphylococcus* | 0.000 |
|  | *Staphylococcus-Pediococcus* | 0.000 |  | *Weissella-Staphylococcus* | 0.005 |
|  | *Latilactobacillus-Levilactobacillus* | 0.009 |  | *Lacticaseibacillus-Enterococcus* | 0.000 |
|  | *Latilactobacillus-Pediococcus* | 0.000 |  | *Lacticaseibacillus-Bifidobacterium* | 0.000 |
|  | *Lactococcus-Levilactobacillus* | 0.000 |  | *Lacticaseibacillus-Staphylococcus* | 0.000 |
|  | *Lactococcus-Pediococcus* | 0.000 |  | *Lactococcus-Enterococcus* | 0.000 |
|  | *Lactobacillus-Levilactobacillus* | 0.000 |  | *Lactococcus-Bifidobacterium* | 0.000 |
|  | *Lactobacillus-Pediococcus* | 0.000 |  | *Lactococcus-Staphylococcus* | 0.000 |
|  | *Lacticaseibacillus-Levilactobacillus* | 0.000 |  | *Levilactobacillus-Bifidobacterium* | 0.000 |
|  | *Lacticaseibacillus-Pediococcus* | 0.000 |  | *Levilactobacillus-Staphylococcus* | 0.000 |
|  | *Lactiplantibacillus-Levilactobacillus* | 0.014 |  | *Leuconostoc-Enterococcus* | 0.000 |
|  | *Lactiplantibacillus-Pediococcus* | 0.000 |  | *Leuconostoc-Bifidobacterium* | 0.000 |
|  | *Limosilactobacillus-Levilactobacillus* | 0.037 |  | *Leuconostoc-Staphylococcus* | 0.000 |
|  | *Limosilactobacillus-Pediococcus* | 0.000 |  | *Lactiplantibacillus-Enterococcus* | 0.000 |
|  | *Enterococcus-Pediococcus* | 0.000 |  | *Lactiplantibacillus-Bifidobacterium* | 0.000 |
|  | *Bifidobacterium-Pediococcus* | 0.000 |  | *Lactiplantibacillus-Staphylococcus* | 0.000 |
|  | *Leuconostoc-Pediococcus* | 0.023 |  | *Latilactobacillus-Bifidobacterium* | 0.001 |
| (B.) **No. of MGEs** | *Lacticaseibacillus-Bifidobacterium* | 0.000 |  | *Latilactobacillus-Staphylococcus* | 0.001 |
|  | *Latilactobacillus-Bifidobacterium* | 0.018 |  | *Lactobacillus-Enterococcus* | 0.002 |
|  | *Leuconostoc-Bifidobacterium* | 0.000 |  | *Lactobacillus-Bifidobacterium* | 0.000 |
|  | *Lacticaseibacillus-Enterococcus* | 0.000 |  | *Lactobacillus-Staphylococcus* | 0.000 |
|  | *Latilactobacillus-Enterococcus* | 0.002 |  | *Lentilactobacillus-Staphylococcus* | 0.026 |
|  | *Leuconostoc-Enterococcus* | 0.000 |  | *Streptococcus-Bifidobacterium* | 0.018 |
|  | *Pediococcus-Enterococcus* | 0.041 |  | *Streptococcus-Staphylococcus* | 0.006 |
|  | *Lactiplantibacillus-Bifidobacterium* | 0.000 |  | *Limosilactobacillus-Bifidobacterium* | 0.002 |
|  | *Lactiplantibacillus-Enterococcus* | 0.000 |  | *Limosilactobacillus-Staphylococcus* | 0.009 |

Statistics: rank analysis of variance (Kruskall-Wallis), pairwise multiple comparisons using Dunn’s test with Bonferroni adjustment; p < 0.05. ARG, antimicrobial resistance gene; MGE, mobile genetic element.
