# Supplementary material for: Lactic acid bacteria and bifidobacteria deliberately introduced into the agro-food chain do not significantly increase the antimicrobial resistance gene pool
Source: Gut Microbes. 2022 Sep 28;14(1):2127438. doi: 10.1080/19490976.2022.2127438 (PMC9542798; doi:10.1080/19490976.2022.2127438)
Supplement: Supplemental Material [file KGMI_A_2127438_SM4927.zip › supplementary.zip!/Supplementary Table S6.docx]

Supplementary Table S6. Selective media, incubation time and temperature, and growth conditions for isolation and propagation of bacterial strains.

|  | Isolation | | | Propagation | | | | Conditions^d^ |
| --- | --- | --- | --- | --- | --- | --- | --- | --- |
|  | Medium^b^ | T^c^ [°C] | Time [h] | | Medium | T [°C] | Time [h] |  |
| Lactobacilli^a^ | MRS | 37 | 72 | | MRS | 37 | 24-72 | anaerobic |
| *Lactiplantibacillus plantarum* | MRS | 30 | 72 | | MRS | 30 | 24-72 | anaerobic |
| *Latilactobacillus* *sakei* | MRS | 30 | 72 | | MRS | 30 | 24-72 | anaerobic |
| *Levilactobacillus brevis* | MRS | 30 | 72 | | MRS | 30 | 24-72 | anaerobic |
| *Lactobacillus acidophilus* group | MRS/CL/CIP^e^ | 37 | 72 | | MRS | 37 | 24-72 | anaerobic |
| *Lacticaseibacillus casei/paracasei/rhamnosus* | MRS | 25/37 | 72 | | MRS | 37 | 24-72 | anaerobic |
| *Pediococcus* sp. | MRS | 37 | 72 | | MRS | 37 | 24-72 | anaerobic |
| *Bifidobacterium* sp. | TOS-MUP^f^ | 37 | 72 | | MRS (+ cys^h^) | 37 | 24-72 | anaerobic |
| *Enterococcus* sp. | M17 | 37 | 24 | | M17 | 37 | 24 | aerobic |
| *Lactococcus* sp. | M17 | 30 | 72 | | M17 | 30 | 24-72 | aerobic |
| *Streptococcus thermophilus* | M17 | 37/45 | 24 | | M17 | 37 | 24 | aerobic |
| *Leuconostoc* sp. | MRS-V^g^ | 30 | 72 | | MRS | 30 | 24-72 | anaerobic |
| *Carnobacterium divergens* | M17 | 30 | 72 | | M17 | 30 | 24-72 | aerobic |
| *Staphylococcus* sp. | M17 | 30 | 24 | | M17 | 30 | 24 | aerobic |

^a^ original genus *Lactobacillus*

^b^ Samples of fermented products were cultured on Rogosa (Merck, Germany), MRS (Merck, Germany), and M17 (Merck, Germany). Rogosa and MRS plates were incubated for 72 hours (37 ˚C or 30 ˚C, anaerobic conditions), M17 plates for 24 hours (30 ˚C, aerobic conditions).

^c^ T, temperature

^d^ Anaerobic conditions were generated using GENbox anaer (BioMérieux, France).

^e^ CL, clindamycin solution (0.2 mg/ml) (Sigma-Aldrich, USA); CIP, ciprofloxacin solution (2 mg/ml) (Fluka, USA)

^f^ 50 mg of mupirocin (MUP) was dissolved in 2.5 ml of 96 % ethanol and added (500 µl) to 200 ml of TOS medium.

^g^ V, vancomycin solution (1 mg/ml) (Sigma-Aldrich, USA)

^h^ cys, L-cysteine hydrochloride solution (0.03 g/ml) (Merck, Germany)
