# Supplementary material for: Lactic acid bacteria and bifidobacteria deliberately introduced into the agro-food chain do not significantly increase the antimicrobial resistance gene pool
Source: Gut Microbes. 2022 Sep 28;14(1):2127438. doi: 10.1080/19490976.2022.2127438 (PMC9542798; doi:10.1080/19490976.2022.2127438)
Supplement: Supplemental Material [file KGMI_A_2127438_SM4927.zip › supplementary.zip!/Supplementary Table S7.docx]

Supplementary Table S7. Species-specific oligonucleotide primers and PCR programs.

| Species | Primer sequence [5' -> 3'] | PCR program^a^ | Length of amplicons [bp] | Source |
| --- | --- | --- | --- | --- |
| 16S rDNA^b^ | 27f: AGAGTTTGATCCTGGCTCAG 1495R: CTACGGCTACCTTGTTACGA | 95°C 60s, 59 °C 60s, 72 °C 120s | 1468 | ^1^ |
| *B. animalis* | B_ani-f: CACCAATGCGGAAGACCAG B_ani-r: GTTGTTGAGAATCAGCGTGG | 95°C 15s, 62 °C 20s, 72 °C 30s | 184 | ^2^ |
| *B. animalis* subsp. *lactis* | BflactF: CCCTTTCCACGGGTCCC BflactR: AAGGGAAACCGTGTCTCCAC | 95°C 15s, 62 °C 20s, 72 °C 30s | 194 | ^3^ |
| *B. longum* | F: CAGTTGATCGCATGGTCTT R: TACCCGTCGAAGCCAC | 95°C 15s, 60 °C 20s, 72 °C 30s | 106 | ^3^ |
| *E. faecalis* | ddl E.faecalis E1: ATCAAGTACAGTTAGTCT  ddl E.faecalis E2: ACGATTCAAAGCTAACTG | 95°C 30s, 55 °C 30s, 72 °C 30s | 941 | ^4^ |
| *E. faecium* | ddl E.faecium F1: AGAGACATTGAATATGCC  ddl E.faecium F2: TCGAATGTGCTACAATC | 95°C 30s, 55 °C 30s, 72 °C 30s | 550 | ^4^ |
| *Lcb. casei/paracasei* 1 | Y2: CCCACTGCTGCCTCCCGTAGGAGT Cas1: TGCACTGAGATTCGACTTAA | 95°C 30s, 62 °C 30s, 72 °C 30s | ~300 | ^5^ |
| *Lcb. casei/paracasei* 2 | Y2: CCCACTGCTGCCTCCCGTAGGAGT Para1: CCGAGATTCAACATGG | 95°C 30s, 55 °C 30s, 72 °C 30s | 290 | ^5^ |
| *Lcb. casei/paracasei/ rhamnosus* multiplex | Czfor: CAGCGCTGGTGGAAGACTTG PC2a: GGATTGGGTTTTGCGTGATGGTCGC Rhfor: GACTTCTCAACCAGCAGCGCAGA CPRev: TGCATTTCCCCGCTTTCATGACT | 95°C 30s, 68 °C 30s, 72 °C 45s | *Lcb. casei* 666 *Lcb. paracasei* 253 *Lcb. rhamnosus* 801 | ^6^ |
| *Lcb. rhamnosus* | PrI: CAGACTGAAAGTCTGACGG RhaII: GCGATGCGAATTTCTATTATT | 95°C 30s, 58 °C 30s, 72 °C 30s | 186 | ^7^ |
| *Lp. plantarum* | Plant 1: ATCATGATTTACATTTGAGTG LOWLAC: CGACGACCATGAACCACCTGT | 95°C 30s, 58 °C 30s, 72 °C 30s | 996 | ^8^ |
| *Lb. acidophilus* | LacI: AGCTGAACCAACAGATTCAC LacII: ACTACCAGGGTATCTAATCC | 95°C 30s, 62 °C 30s, 72 °C 30s | 759 | ^7^ |
| *Lb. delbrueckii* subsp. *bulgaricus* | LB1: AAAAATGAAGTTGTTTAAAGTAGGTA LLB1: AAGTCTGTCCTCTGGCTGG | 95°C 30s, 58 °C 30s, 72 °C 30s | 1065 | ^9^ |

| Species | Primer sequence [5' -> 3'] | PCR program^a^ | Length of amplicons [bp] | Source |
| --- | --- | --- | --- | --- |
| *Lb. gasseri* | Gas I: GAGTGCGAGAGCACTAAAG Gas II: CTATTTCAAGTTGAGTTTCTCT | 95°C 30s, 55 °C 30s, 72 °C 30s | 197 | ^7^ |
| *Lb. johnsonii* | Ljo I: GAGCTTGCCTAGATGATTTTA Ljo II: ACTACCAGGGTATCTAATCC | 95°C 30s, 57 °C 30s, 72 °C 30s |  | ^7^ |
| *Lc. lactis* | 27f: AGAGTTTGATCMTGGCTCAG Lla: CAGTCGGTACAAGTACCAAC | 95°C 30s, 55 °C 30s, 72 °C 30s | 87 | ^10^ |
| *La. curvatus* | 16: GCTGGATCACCTCCTTTC Lc: TTGGTACTATTTAATTCTTA | 95°C 30s, 53 °C 30s, 72 °C 30s | 220 | ^11^ |
| *La. sakei* | 16: GCTGGATCACCTCCTTTC Ls(/16): /ATGAAACTATTAAATTGGTAC | 95°C 30s, 53 °C 30s, 72 °C 30s | 220 | ^11^ |
| *Le. mesenteroides* | Lmes-f: AACTTAGTGTCGCATGAC Lmes-r: AGTCGAGTTACAGACTACAA | 95°C 60s, 60 °C 60s, 72 °C 120s | 1150 | ^12^ |
| *Lv. brevis* | BREVIS-F: CTTGCACTGATTTTAACA BREVIS-R: GGGCGGTGTGTACAAGGC | 95°C 30s, 40 °C 30s, 72 °C 60s | 1340 | ^13^ |
| *Lg. salivarius* | Sal1: ATTCACTCGTAAGAAGT LOWLAC: CGACGACCATGAACCACCTGT | 95°C 30s, 50 °C 30s, 72 °C 60s | 993 | ^8^ |
| *Lm. fermentum* | Lfpr: GCCGCCTAAGGTGGGACAGAT FermII: CTGATCGTAGATCAGTCAAG | 95°C 30s, 55 °C 30s, 72 °C 30s | ~250 | ^7^ |
| *Lm. reuteri* | Lfpr: GCCGCCTAAGGTGGGACAGAT Reu: AACACTCAAGGATTGTCTGA | 95°C 30s, 55 °C 30s, 72 °C 30s | ~250 | ^7^ |
| *P. acidilactici* | PAC23S_F: GTTTCGGAGGAGGCGCAA P23S_R: CTGTCTCGCAGTCAAGCTC | 95°C 30s, 66 °C 60s, 72 °C 60s | 213 | ^14^ |
| *P. pentosaceus* | PPE23S_F: CCAGGTTGAAGGTGCAGTAAAAT P23S_R: CTGTCTCGCAGTCAAGCTC | 95°C 30s, 66 °C 30s, 72 °C 60s | 1647 | ^14^ |
| genera *Staphylococcus* | STA I: GGAATAACGTGACATATTGTA STA II: TTCACTCGGTTTTGCTTGG | 95°C 30s, 55 °C 30s, 72 °C 30s | 100-200 | ^15^ |
| *S. thermophilus* | Th I: ACGGAATGTACTTGAGTTTC Th II: TTTGGCCTTTCGACCTAAC | 95°C 30s, 58 °C 30s, 72 °C 30s | 205-304 | ^16^ |

*La., Latilactobacillus; Lb., Lactobacillus; Lc., Lactococcus; Lcb., Lacticaseibacillus; Le., Leuconostoc; Lg., Ligilactobacillus; Lm., Limosilactobacillus; Lp., Lactiplantibacillus; Lv., Levilactobacillus; P., Pediococcus; S., Streptococcus.*

^a^ PCR reaction mixtures consisted of 1× PCR buffer Go Taq flexi (Promega, USA), 1.5 mM MgCl2, 0.025 U/µl Taq polymerase, 0.1 mM dNTP (Thermo Fisher Scientific, USA), and 0.5 mM of each primer. PCR was performed with an initial denaturation at 95 °C for 180 s, followed by 30 cycles under the conditions indicated above, and a final extension cycle at 72 °C for 300 s.

^b^ The 16S rDNA amplicons were purified using the PureLink™ Quick gel extraction and PCR purification Combo Kit (Invitrogen, USA) or the Wizard® SV Gel and PCR Clean-Up System (Promega, USA).

**References**

1. Yu J, Wang WH, Menghe BLG, Jiri MT, Wang HM, Liu WJ, Bao QH, Lu Q, Zhang JC, Wang F, et al. Diversity of lactic acid bacteria associated with traditional fermented dairy products in Mongolia. Journal of Dairy Science. 2011;94(7):3229–3241. doi:10.3168/jds.2010-3727

2. Junick J, Blaut M. Quantification of human fecal *Bifidobacterium* species by use of quantitative real-time PCR analysis targeting the *groEL* gene. Applied and Environmental Microbiology. 2012;78(8):2613–2622. doi:10.1128/AEM.07749-11

3. Malinen E, Kassinen A, Rinttilä T, Palva A. Comparison of real-time PCR with SYBR green I or 5′-nuclease assays and dot-blot hybridization with rDNA-targeted oligonucleotide probes in quantification of selected faecal bacteria. Microbiology. 2003;149:269–277. doi:10.1099/mic.0.25975-0

4. Dutka-Malen S, Evers S, Courvalin P. Detection of glycopeptide resistance genotypes and identification to the species level of clinically relevant enterococci by PCR. Journal of Clinical Microbiology. 1995;33:24–27. doi:10.1128/jcm.33.5.1434-1434.1995

5. Ward LJH, Timmins MJ. Differentiation of *Lactobacillus casei, Lactobacillus paracasei* and *Lactobacillus rhamnosus* by polymerase chain reaction. Letters in Applied Microbiology. 1999;29(2):90–92. doi:10.1046/j.1365-2672.1999.00586.x

6. Bottari B, Felis GE, Salvetti E, Castioni A, Campedelli I, Torriani S, Bernini V, Gatti M. Effective identification of *Lactobacillus* *casei* group species: genome-based selection of the gene *mutL* as the target of a novel multiplex PCR assay. Microbiology. 2017;163(7):950–960. doi:10.1099/mic.0.000497

7. Walter J, Tannock GW, Tilsala-Timisjarvi A, Rodtong S, Loach DM, Munro K, Alatossava T. Detection and identification of gastrointestinal *Lactobacillus* species by using denaturing gradient gel electrophoresis and species-specific PCR primers. Applied and Environmental Microbiology. 2000;66:297–303. doi:10.1128/AEM.66.1.297-303.2000

8. Chagnaud P, Machinis K, Coutte LA, Marecat A, Mercenier A. Rapid PCR-based procedure to identify lactic acid bacteria: application to six common *Lactobacillus* species. Journal of Microbiological Methods. 2001;44(2):139–148. doi:10.1016/S0167-7012(00)00244-X

9. Torriani S, Zapparoli G, Dellaglio F. Use of PCR-based methods for rapid differentiation of *Lactobacillus* *delbrueckii* subsp. *bulgaricus* and *L.* *delbrueckii* subsp. *lactis*. Applied and Environmental Microbiology. 1999;65(10):4351–4356.

10. Barakat RK, Griffiths MW, Harris LJ. Isolation and characterization of *Carnobacterium*, *Lactococcus*, and *Enterococcus* spp. from cooked, modified atmosphere packaged, refrigerated, poultry meat. International Journal of Food Microbiology. 2000;62:83–94. doi:10.1016/S0168-1605(00)00381-0

11. Berthier F, Ehrlich SD. Genetic diversity within *Lactobacillus* *sakei* and *Lactobacillus* *curvatus* and design of PCR primers for its detection using randomly amplified polymorphic DNA. International Journal of Systematic Bacteriology. 1999;49:997–1007. doi:10.1099/00207713-49-3-997

12. Lee HJ, Park SY, Kim J. Multiplex PCR-based detection and identification of *Leuconostoc* species. FEMS Microbiology Letters. 2000;193(2):243–247. doi:10.1016/S0378-1097(00)00489-4

13. Guarneri T, Rossetti L, Giraffa G. Rapid identification of *Lactobacillus* *brevis* using the polymerase chain reaction. Letters in Applied Microbiology. 2001;33(5):377–381. doi:10.1046/j.1472-765X.2001.01014.x

14. Pfannebecker J, Fröhlich J. Use of a species-specific multiplex PCR for the identification of pediococci. International Journal of Food Microbiology. 2008;128(2):288–296. doi:10.1016/j.ijfoodmicro.2008.08.019

15. Forsman P, Tilsala-Timisjärvi A, Alatossava T. Identification of staphylococcal and streptococcal causes of bovine mastitis using 16S-23S rRNA spacer regions. Microbiology. 1997;143:3491–3500. doi:10.1099/00221287-143-11-3491

16. Tilsala-Timisjärvi A, Alatossava T. Development of oligonucleotide primers from the 16S-23S rRNA intergenic sequences for identifying different dairy and probiotic lactic acid bacteria by PCR. International Journal of Food Microbiology. 1997;35:49–56. doi:10.1016/S0168-1605(97)88066-X
